# Exposome and Trans-syndromal Developmental Trajectories Toward Psychosis

**DOI:** 10.1016/j.bpsgos.2022.05.001

**Published:** 2022-05-25

**Authors:** Ran Barzilay, Lotta-Katrin Pries, Tyler M. Moore, Raquel E. Gur, Jim van Os, Bart P.F. Rutten, Sinan Guloksuz

**Affiliations:** a Department of Psychiatry, Perelman School of Medicine, University of Pennsylvania, Philadelphia, Pennsylvania; b Lifespan Brain Institute of the Children’s Hospital of Philadelphia and Penn Medicine, Philadelphia, Pennsylvania; c Department of Child and Adolescent Psychiatry and Behavioral Science, Children’s Hospital of Philadelphia, Philadelphia, Pennsylvania; d Department of Psychiatry, Yale University School of Medicine, New Haven, Connecticut; e Department of Psychiatry and Neuropsychology, School for Mental Health and Neuroscience, Maastricht University Medical Center, Maastricht, The Netherlands; f Department of Psychiatry, UMC Utrecht Brain Center, University Medical Center Utrecht, Utrecht University, Utrecht, The Netherlands; g Department of Psychosis Studies, Institute of Psychiatry, Psychology & Neuroscience, King’s College London, London, United Kingdom

**Keywords:** Environment, Genetics, Neurodevelopment, Schizophrenia, Transdiagnostic, Youth mental health

## Abstract

The prenatal period, early childhood, and adolescence are considered sensitive periods for brain and behavior development, when environmental exposures may have long-lasting effects on mental health. Psychosis spectrum disorder (PSD) is a developmental disorder that often manifests with nonspecific clinical presentations long before full-blown PSD is diagnosed. Genetic factors only partly explain PSD. Multiple early-life environmental exposures are associated with PSD. In this review, we describe the conceptual framework of the exposome and its relevance to PSD research in developmental cohorts and beyond and discuss key challenges for the field as it attempts to move beyond studying environment (in the sense of “searching under the lamppost because this is where the light is”) to a more comprehensive assessment of environment and its contribution to PSD. We then suggest that the field should aspire to studying environmental origins of PSD through a developmental lens focusing on young cohorts and using multilevel phenotyping of environment, adopting an exposome framework that embraces the dynamic complex nature of environment and acknowledges the effect of additive and interactive environmental exposures alongside the genome. Furthermore, we highlight the need for a developmental perspective when studying exposome effects on psychopathology, accepting the nonspecificity of child/adolescent psychopathology and encouraging the study of trans-syndromal manifestations, shifting the research paradigm from categorical outcomes (e.g., schizophrenia) and going beyond clinical settings to investigate trajectories of risk and resilience.

“Science is made up of so many things that appear obvious after they are explained” (1).

Psychosis spectrum disorder (PSD) is a heterogeneous and multidimensional phenotype with a multifactorial etiology (2). Evidence suggests that PSD is a neurodevelopmental condition (3), with deviance from the typical neurodevelopmental trajectory often observed around adolescence and young adulthood. The genetic component of PSD involves a few very rare variants with moderate effect sizes, but primarily many small-effect-size common variants (single nucleotide polymorphisms) that can be quantified as a polygenic risk score for schizophrenia (SCZ) (4). Although previous family and twin studies have suggested around 60% to 80% heritability (5), the single nucleotide polymorphism–based heritability estimated in the recent Psychiatric Genomics Consortium SCZ genome-wide association study (GWAS) was 0.24, with polygenic risk score for SCZ explaining only 0.077 of the variance in liability (6). Building off the accumulating data suggesting that increased GWAS sample size increases the variance explained in the phenotype (7), it is likely that genomic data will explain more variance in PSD as larger GWASs become available. However, given that the latest SCZ GWAS of 76,555 patients with SCZ and almost 250,000 control subjects allowed calculation of a polygenic risk score that explains <8% of the variance (6), and even when considering the contribution of rare genetic variations and of gene × gene interaction effects underlying psychosis liability, it is fair to assume that the contribution of nongenetic factors (i.e., environment) to PSD is substantial.

## Environmental Research in Psychosis

Over the last 2 decades, hypothesis-driven research into environmental factors contributing to PSD has identified several parameters associated with psychosis at varying evidence levels and consistency across studies (8). Some of these environmental factors include cannabis use, childhood traumatic experiences, pre- and perinatal adversity, ethnic minority and migration status, vitamin D deficiency, urbanicity, tobacco smoking, winter birth, exposure to peer bullying, and air pollution (9,10). Notably, most environmental exposures associated with PSD risk are experienced early in the lifespan (11) during sensitive periods of brain development (12). The multiplicity of exposures and the likely critical role of timing clearly pose a major challenge for elucidating the specific and collective contribution of environmental factors to psychosis development. The level of complexity calls for a paradigm shift, similar to the agnostic GWAS replacing candidate gene studies in psychiatric genomics.

## Moving From Single Exposures to the Burden of Multiple Exposures

**Figure 1:**
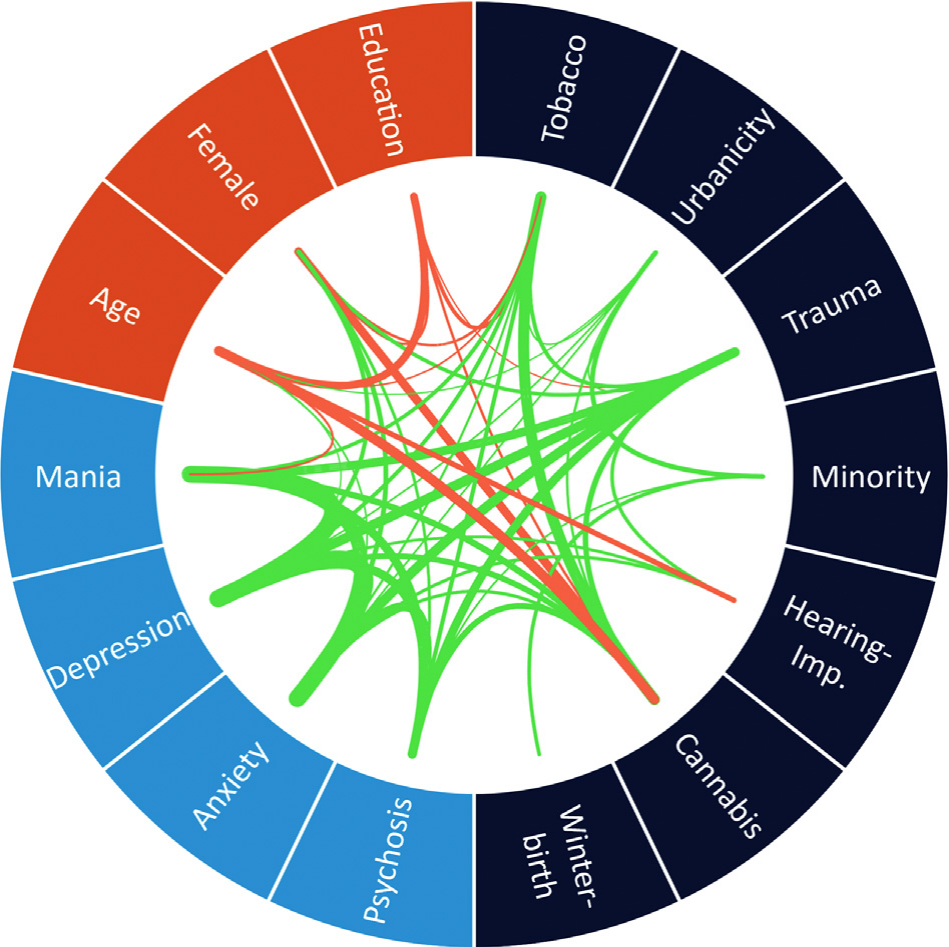
Correlation globe of exposures in the Netherlands Mental Health Survey and Incidence Study-2 (13). Only nominally statistically significant (*p* < .05) correlations are shown. Line thickness marks the magnitude of the correlation. Green lines indicate positive correlations, and red lines indicate negative correlations. Hearing-Imp., hearing impaired.

The complexity of certain characteristics of components of the environment is similar to that of genetics. Most exposures are highly correlated, pleiotropic, and interactive (13). The dynamic nature of the environment adds another layer of challenge when investigating the effects of the onset, duration, periodicity, and severity of exposure on mental health outcomes over a lifespan. Furthermore, accumulating evidence provides support for the stress-vulnerability model (or diathesis-stress model) (14) of psychosis (and of psychopathology generally), such that genes influence environmental sensitivity (gene × environment interaction), genes influence exposure to environment in the causal path to mental illness, or genes contribute to mental illness and environmental exposure independently (environment not in the causal path, i.e., gene-environment correlations). It is clear that the contribution of environment to psychosis cannot be fully understood when exposures are investigated in isolation. Figure 1 illustrates the level of complexity when environmental factors for psychosis, demographic characteristics, and clinical outcomes are interconnected (13).

To move the field forward, we recently proposed that psychiatric research needs to adopt the exposome framework to embrace the complex network of exposures and to understand the role of environment (15,16). The exposome represents the totality of exposures (nongenetic component) in an individual’s lifetime and consists of three domains: general external (e.g., urbanicity), specific external (e.g., pollutants), and internal (e.g., gut microbiota) (17).

In this review, we will summarize the challenges of investigating the role of the exposome in the multidimensional psychosis phenotype. We will attempt to make a case for the need for a developmental framework that adopts a trans-syndromal—i.e., agnostic to diagnoses versus a transdiagnostic framework that intrinsically necessitates diagnoses—dimensional approach to understanding the role of the exposome in developmental psychopathology broadly, including but not limited to psychosis. Finally, we will discuss future directions focusing on key open questions for the field of exposomes in psychiatric research.

## Environment in Early Life, From Pregnancy to Adolescence, Is Most Critical

**Figure 2:**
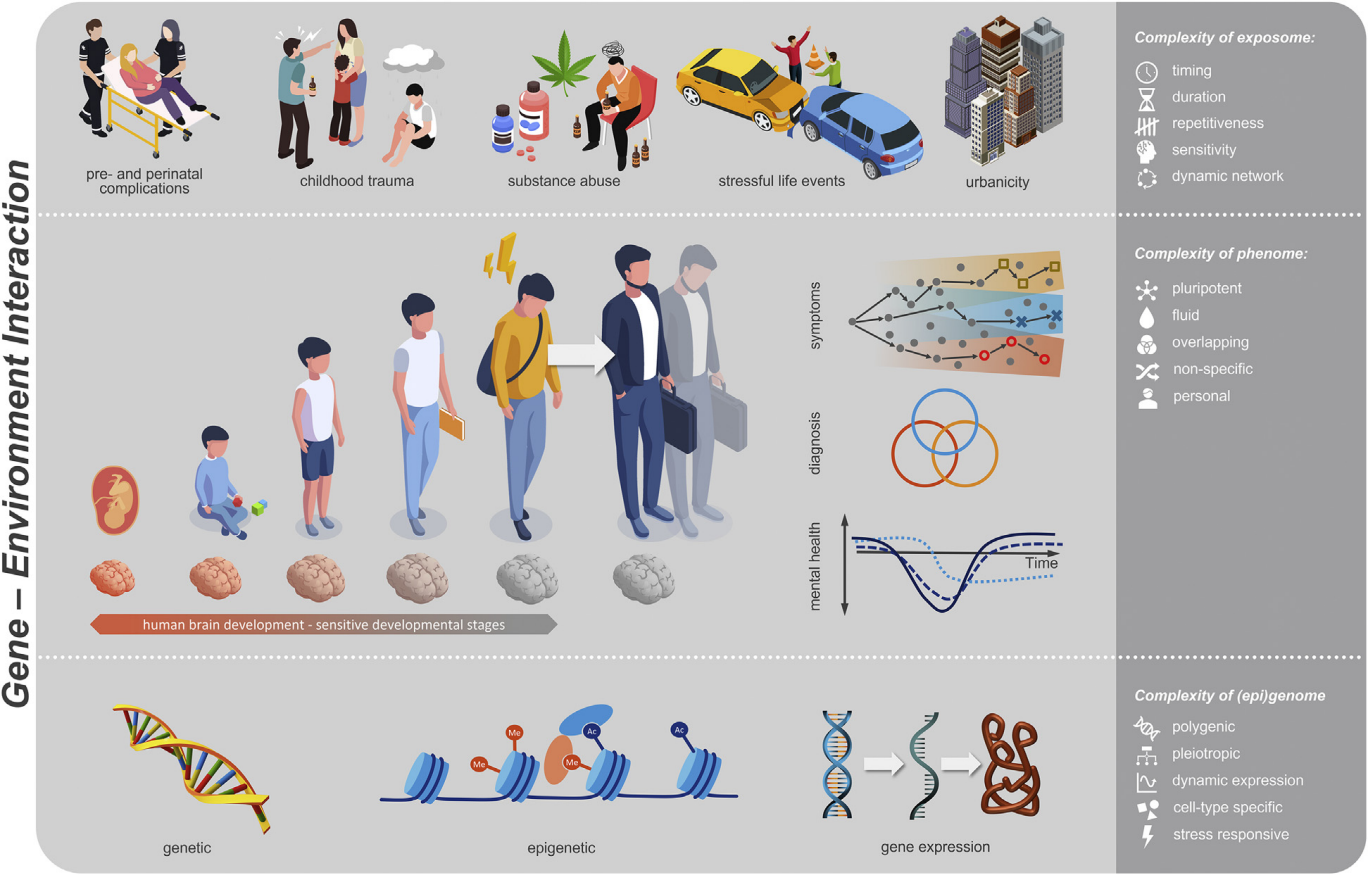
Challenges of investigating the complex multifactorial etiology of mental health trajectories involving an interplay of exposome, genome, and epigenome.

PSD is recognized as neurodevelopmental (3), and many of the environmental exposures associated with increased risk of PSD occur in childhood and adolescence (18). Specific focus has been given to exposures very early in the lifespan, around pregnancy or birth (19), and during adolescence (20). It is believed that these two life periods, at which brain maturation peaks, are especially sensitive to environmental cues impacting on the brain and its development (12). Figure 2 visualizes the complexities of the phenome, exposome, and genome across early development.

Studies in animal models (21,22) and clinical/epidemiological human research (23, 24, 25) have consistently shown that occurrence of environmental stress during a sensitive period may have long-lasting implications for brain development and function that may manifest in psychiatric symptomatology into late adulthood. Specifically in the context of PSD, one mechanism that ties environmental exposures in a sensitive period with risk is that of disrupted synaptic pruning during adolescence (26). Findings in genetic studies in patients with PSD and their consequent validation in animal models (27) and patient-derived cellular models (28) converge to suggest the involvement of immune activation that drives abnormal synaptic pruning. A key challenge for future exposome research in a neurodevelopmental context would be considering the timing of exposures and evaluating their long-term consequences on brain and behavior. While these types of studies are difficult to achieve in human samples because of the temporal granularity of exposure data, it is likely that animal models of prenatal stress (e.g., immune activation) can help close some of the gaps linking the timing of exposures, biological mediators of exposures (e.g., inflammation), and later-life brain and behavior function (29).

However, research also suggests that there is another side to the coin of environmental exposures during sensitive periods, and it is likely that exposures to positive environmental factors (or potentially removal of adverse exposures) during sensitive periods will be associated with reduced long-term psychiatric risk and that exposures to fostering environments at sensitive periods may enhance resilience (30). The challenge is therefore to identify environmental exposures early in the lifespan that can be linked to increased or decreased psychosis risk and that can theoretically be modified to shift the trajectory from that of risk to that of resilience (31). It is assumed that resilience, similar to environmental stress–imposed risk, is a dynamic complex process that integrates multiple developing systems (32,33). Evidence suggests that environmental factors such as family (34), school (35), and neighborhood (36) environments are critical to allowing adaptive behavior in the face of developmental stress. In the context of PSD, recent works attempt to adopt this conceptual framework of incorporating social environmental factors when modeling risk and resilient trajectories of psychosis onset (37). Therefore, parallel to studies of environmental risk factors, effort should be made to investigate how environmental context contributes to resilient outcomes.

## Exposures Are Interdependent

Studies suggest that early-life environmental adversities have a role in PSD onset, while also highlighting the challenge of dissecting the specificity of environment effects. A good example in the field of psychosis research is the finding of urbanicity as a risk factor for SCZ (38). It is likely that this elusive term “urbanicity” reduces dimensionality of multiple specific exposome features (e.g., air pollution, seasonal viruses, city-life stress). A major challenge for our field is to try to address the specificity of environmental effects that are highly collinear and often co-occur (13). For example, trauma is often associated with poverty, is more likely to occur in urban settings, and is associated with higher likelihood of fewer educational resources and less access to mental health care, all key risk factors for developmental psychopathology. Similarly, prenatal exposure to cannabis is likely collinear with prenatal exposure to other substances, including tobacco and alcohol (39). In a broader context, maternal prenatal use of substances is likely collinear with lower levels of education and income and lower access to prenatal care and pediatric care thereafter. The question then becomes, when using a hypothesis-driven framework to test associations between trauma and psychosis or between prenatal cannabis exposure and psychosis, whether and how to specifically test associations of the index exposure of interest with the outcome (i.e., psychosis). In that context, these data should encourage researchers studying environmental effects on neurodevelopment to look further than “under the lamppost” and to address the problem of collinearity, accepting that it is the rule rather than the exception. We therefore suggest that such an exposome approach is warranted, whereby environmental exposures should be studied by accounting for their collinear nature. We argue that this framework better captures real life, where context of exposure may be key in determining the long-lasting effect on the developing brain, as was shown in animal models (22).

## Predictive Modeling Approach for Estimating Exposome Score for SCZ

In accordance with the liability-threshold model, the combination of environmental and genetic factors increases the liability to psychosis additively (40). Recently, there have been increasing efforts to estimate an environmental liability score for SCZ (41). We constructed the exposome score for SCZ (ES-SCZ) that consisted of previously studied environmental factors associated with psychosis, including 5 domains of childhood adversities (emotional, sexual, and physical abuse along with emotional and physical neglect), bullying, cannabis use, winter birth, and hearing impairment (42). This single metric score has 2 advantages. First, ES-SCZ uses weighted effect sizes for each exposure and therefore provides a better estimate than the simple exposure counting approach that assumes equal risk per exposure. Second, the weighted effect sizes of ES-SCZ are derived from a model that takes into account interdependency of exposures and therefore performs better in predicting psychosis than the sum score and the environmental score derived from meta-analytic estimates (43). We have investigated the predictive performance and the utility of ES-SCZ in the general population cohort and independent samples of individuals with PSD (Table 1). Overall, these studies show that ES-SCZ, an index of environmental predisposition, can be used to test gene × environment (44) and environment × environment (45) interactions, improve psychosis prediction and risk stratification in the general population (43,46), and enhance clinical outcome prognostication in individuals with PSD (47,48). ES-SCZ offers a practical and consistent solution that can be applied across independent samples (given consistent collection of exposures across samples) to yield comparable findings. With an increasing availability of rich environmental datasets and an ever-expanding knowledge base, more environmental factors, such as cyberbullying and pre- and perinatal complications, may be added to ES-SCZ for further improvement.

**Table 1:** Overview of Studies That Used ES-SCZ

Reference	Study Population	Key Findings
Pries *et al.*, 2019 (42)	Multinational study of individuals with SCZ, unaffected siblings, and control subjects (EUGEI and GROUP)	ES-SCZ performed better than environmental scores assuming independent effects (e.g., Emet, Esum) ES-SCZ significantly distinguished three groups: ES-SCZ of patients > siblings > healthy control subjects The odds of SCZ risk increased incrementally as a function of ES-SCZ
Guloksuz *et al.*, 2020 (46)	A population-based prospective cohort study in the Netherlands (NEMESIS-2)	ES-SCZ was associated with five levels of psychosis risk strata (no risk as the reference group) ES-SCZ differentiated risk strata: moderate risk > low risk; high risk > low risk; and clinical psychosis > low risk
Pries *et al.*, 2020a (44)	Multinational study of individuals with SCZ, unaffected siblings, and control subjects (EUGEI and GROUP)	Significant main and interacting associations of ES-SCZ and PRS-SCZ with case-control status Significant main and interacting associations of ES-SCZ and PRS-SCZ with schizotypy in control subjects and siblings
Pries *et al.*, 2020b (45)	A population-based prospective cohort study in the Netherlands (NEMESIS-2)	Significant main and interacting associations of ES-SCZ and recent stressful life events with poor mental and physical health outcomes
Pries *et al.*, 2021 (43)	A population-based prospective cohort study in the Netherlands (NEMESIS-2)	ES-SCZ showed a significantly better discriminative function (AUC = 0.84; LR^−^ = 0.20; LR^+^ = 3.86) than Emet and Esum ES-SCZ was associated with SCZ diagnosis with the highest OR (2.76 [95% CI = 2.20 – 3.46]) and the greatest explained variance (*R*^2^ = 14.03%) among 33 mental and physical health outcomes
Erzin *et al.*, 2021a (48)	Multinational study of individuals with SCZ, unaffected siblings, and control subjects (EUGEI and GROUP)	ES-SCZ was associated with poor global functioning domains in control subjects, siblings, and patients in EUGEI, also after controlling for PRS-SCZ Results were replicated independently in GROUP
Erzin *et al.*, 2021b (47)	One-year follow-up study of individuals with FEP in Greece (Athens FEP Research Study)	ES-SCZ was associated with poor global and specific functioning domains at baseline and 1 month, also after controlling for demographic, familial, and other environmental factors and clinical features ES-SCZ predicted poor improvement in symptom severity (particularly negative symptoms) from baseline to 1 month assessment

AUC, area under the receiver operating characteristic curve; Emet, aggregate environmental score weighted by the meta-analytic estimates; Esum, environmental sum score; ES-SCZ, exposome score for schizophrenia; EUGEI, European Network of National Networks studying Gene-Environment Interactions in Schizophrenia; FEP, first-episode psychosis; GROUP, Genetic Risk and Outcome of Psychosis Study; LR, likelihood ratio; NEMESIS-2, Netherlands Mental Health Survey and Incidence Study-2; OR, odds ratio; PRS-SCZ, polygenic risk score for schizophrenia; SCZ, schizophrenia.

## The Potential of a Data-Driven Approach to Capture the Exposome

To capture the exposome in early adolescence, we recently applied an approach using dimensionality reduction and bifactor modeling of the exposome in 2 youth cohorts (the Philadelphia Neurodevelopmental Cohort [PNC] and the Adolescent Brain Cognitive Development [ABCD] Study), showing that such an approach indeed facilitates generalizability across cohorts (49). Briefly, we combed through each cohort’s data and reduced dimensionality of all environmental exposures (798 exposures in the ABCD Study and 29 exposures in PNC) to a limited set of correlated environmental factor scores that capture different levels of exposure (e.g., household, neighborhood). Thereafter, we estimated a bifactor model that yields a general environmental score (i.e., ES) that is orthogonal to the domain-specific environmental factor scores. The general ES receives loadings from all environmental exposures included in the model and therefore represents a weighted sum of multilevel environmental exposures for each study participant. After calculating the general exposome factor in the 2 independent datasets, we have shown that the general ES is associated with youth mental and general health measures in an almost identical magnitude (i.e., effect size), despite the fact that ABCD Study and PNC data were collected in different places and at different times with substantial differences in environmental phenotyping (49). We suggest that this general ES can potentially be useful because it captures a latent environment factor that is less likely to be skewed by a specific environmental exposure and might allow for better generalization of environment modeling across different cohorts, which is often very different. In addition, in this issue, we describe a study (50) that dissects the association between psychotic experiences and 6 exposome domains (household adversity, neighborhood environment, day-to-day experiences, state-level environment, family values, pregnancy/birth complications) in the ABCD Study. Our findings suggest that psychotic experiences were particularly associated with the following exposome factor scores: household adversity, day-to-day experiences, and pregnancy/birth complications.

Notably, in addition to the notion that accumulation of environmental stress contributes to disease liability, it is important to consider the context of environmental exposures, whereby individual-level and group-level (e.g., race, ethnicity) exposures to adversity are related to, and likely interact with, the neighborhood and more distal societal environment (as summarized recently in the context of psychosis risk) (37,51). In accordance with the diathesis-stress theory, evidence suggests that the environmental factors interact with each other (environment × environment). Our findings demonstrated that ES-SCZ (an index of environmental predisposition to psychosis) increased the association of recent stressful life events (a precipitating environmental stressor) with mental and physical health outcomes in the general population (45). In addition, we have recently shown in the PNC that neighborhood socioeconomic status environment moderates the association of trauma exposure with suicidal ideation in youth (52). We anticipate that with the growing availability of large datasets with deep phenotyping of the exposome, novel analytic methods will allow teasing apart significant interactions among different exposures at different developmental stages in life.

## Environment Is Rarely Specific to Early Psychopathology That Is Characterized by Heterotypic Continuity

The previous sections addressed the challenge of specificity from the exposure side, yet when studying developmental psychopathology, we must address the challenge of specificity of the outcome (or trajectory, i.e., dependent variable). Evidently, clinical psychiatric manifestations in youth are highly heterogeneous and nonspecific (53) and are characterized by heterotypic continuity, whereby early presentations of symptoms may fully progress into a distinct disorder of different symptom domains later in the developmental course (54). For example, when studying psychosis, it is often preceded by a nonspecific prodrome that can manifest in milder psychopathology than psychosis with several comorbid domains (55), including externalizing, inattention, anxiety, and mood. Similarly, psychotic symptoms in childhood and adolescence may be transient (56). In addition, when studying teens, it is critical to consider the context of adolescence, a developmental phase that is characterized by frequent fluctuations in mood and affect, changes in sleep patterns, and risky behaviors, all considered hallmark psychopathology symptoms.

The challenge therefore is to try to identify exposures that bear specific risk to develop phenotype A and not B. In the context of psychosis, potential research paradigms that can unravel specific exposome effects in psychosis may involve studying the exposome in a population that is at increased risk for psychosis, such as those with a family history of SCZ or those with genetic susceptibility such as a 22q deletion, and identify exposures that are more prevalent in individuals with PSD versus those without PSD. Alternatively, addressing specificity from exposure to psychosis can also be tested to a degree when controlling for nonpsychosis psychopathology, for example, by contrasting effects of the exposome on PSD compared with effects on general psychopathology that accounts for all psychopathology, such as the P-factor.

## Exposome as the Potential Key to Parse Heterogeneity in Neurodevelopmental Trajectories

The growing realization that the developmental trajectory of psychosis is highly variable (as in other developmental psychopathology dimensions) has led the field from the conceptualization of risk as “psychosis ultra-high risk” to a more nonspecific “pluripotent ultra-high risk” for psychiatric outcomes (57,58). Little is known regarding what makes some ultra-high risk youth resilient and bounce back toward typical development, while a minority of 15% will develop full-blown psychosis and some will develop nonpsychotic psychiatric illnesses (59). While genetic susceptibility likely contributes to this heterogeneity, we postulate that the contribution of the exposome is key to understanding a major part of this variance. Notably, addressing the challenges of specificity described above can allow identification of modifiable targets for intervention within the exposome that can shift the developmental trajectory from risk to resilience. Below, we present some key questions for the field that we suggest can advance the mechanistic understanding of the exposome’s role in neurodevelopmental conditions such as psychosis.

## Key Open Questions and a Tentative Road Map for Future Directions for the Field

The exposome paradigm is gaining traction in medical research with increasing interest for its role in mental health outcomes (16). Still, as a young field, some key questions remain that seem pertinent when applying the exposome framework in developmental psychopathology research.

### Is There a Gold Standard to Model the Exposome in Psychiatric Research?

It is well accepted that environment should be examined in a broad context when studying development (60). Still, when studying environment’s effect (more likely association) on brain (structure and function) and behavior outcomes in youth cohorts, different research groups use different (often arbitrary) measures or proxies of environment. Examples include but are not limited to parental education or income to model socioeconomic status, some geocoded measures to model neighborhood environment, different measures of trauma exposure (either self-report or parent report), and other anecdotal exposures that may be relevant to the study question. These measures of environment also are often used as covariates in models and are often inconsistent across studies. We propose that this lack of consistency may explain some of the replication crisis in our field (61). We suggest that a critical step to increase generalizability of findings is for the field to aspire to reach a consensus regarding which factors are needed to be controlled for (as potential confounders). We further suggest that until that consensus is reached, researchers should attempt to account for as many environmental measures as are available (accounting for their collinearity), test how these affect their findings, and to report this to enhance replicability.

### Are There Specific Environmental Domains That Are Especially Critical to Explore?

As described above, multiple environmental exposures have been linked with adverse mental health outcomes, and these were shown at many levels of exposure (9,10). A key unexplored domain that is highly relevant for youth is the virtual environment (i.e., the digital life environment), which will likely contribute to explaining variance in mental health development in youth as more data become available (62). Indeed, when we consider peer victimization as a critical exposure during childhood/adolescence, existing data support the notion that cybervictimization may have comparable detrimental effects to offline peer victimization on youth mental health, including psychosis (63). Notably, the virtual environment is expected to play an increasing role in youth’s mental health following the social changes brought by the COVID-19 pandemic, which include increased screen time and change in social interactions (64). Taken together, future studies on digital life exposures can further delineate exposome effects on youth mental health.

Besides the need for data on the virtual environment, we suggest that it is not likely that the breakthrough in environmental research on mental health development will come from studying unexplored environmental domains; rather, we suggest that the integration of multiple levels of exposures (additive environmental effects or environmental × environmental interaction) can enhance our understanding of the exposome’s role in mental health development. In that context, youth cohorts such as the ABCD Study are likely to advance the field owing to the multilevel phenotyping of environment that spans exposures from individual to state level (65).

### How Critical Is Harmonization in Measurement of the Environment Across Cohorts?

While ideally the field should aspire to harmonize exposome measurement across datasets, we acknowledge that perfect harmonization across cohorts globally may not be practical because of differences between cultures, populations, and research resources (51). Therefore, use of existing (already collected) datasets can try to address the question of what allows generalization even in cases in which measurement of environmental exposures is very different between cohorts. A key challenge is to develop methods that allow quantification of an environmental loading that can be derived from different measurements across cohorts but can still capture an overlapping latent exposome construct. Because the exposome is the entirety of exposures in a lifetime, population-based birth cohort studies covering the neurodevelopmental period, as well as the onset and progression of mental disorders, are required to understand the role of environment in psychopathology. We also suggest that a good rule of thumb when collecting exposome data in cohorts is to characterize environment on multiple levels of exposure, using data collected from multiple sources, such as child report of individual experiences, parent/child report on household/family environment, data from educational environment (schools), data from neighborhood including social exposures (e.g., crime, population density), geographic information system databases, and chemical exposures (e.g., pollutant levels). These multidimensional data will likely decrease measurement error and enhance generalization across cohorts.

### How Stable Is the ES Throughout the Lifespan?

Is the ES closer to genomic scores (i.e., fixed), or is the ES more dynamic (e.g., unexpected trauma that changes dramatically the child’s trajectory if it hits in a sensitive period with lack of buffering/resilient factors)? Addressing this question seems pivotal to determining study designs and modeling of longitudinal data, especially in light of emerging evidence for the importance of predictability (or lack thereof) of environmental factors and detrimental effect of environmental factors on development (66). It is critical that research on environment captures this dimension of consistency/unpredictability to explain variance in trajectories. In addition, this question has implications when modeling the exposome in longitudinal studies. The field needs to agree how to best model the exposome over time. Is it an accumulation of exposures over time (i.e., allostatic load model) (67), or should we model longitudinal trajectories of the exposome?

### How Can We Distinguish Exposome From Genome (and Should We Even Attempt That)?

With the expanding knowledge of gene-environment correlations and gene × environment effects, it becomes clearer that genomic liability and exposomic liability cannot be fully teased apart (68). The question then becomes where the exposome ends and what can be defined as strictly genetic. For example, when studying youth cohorts, should parental psychopathology be considered as exposomic? Another challenge is how to design analyses that capitalize on genetically informed datasets (e.g., twin and other kinship designs, polygenic scores) to model the genome and exposome relationship in a manner that maximizes the ability to explain variance in developmental trajectories. Notably, recent research indeed suggests that environmental insults can unmask genetic vulnerability for PSD risk (69), encouraging the field to incorporate gene and environment factors when modeling PSD risk. Finally, more studies are needed to address the epigenetic effects of environment, because epigenetic mechanisms likely mediate the relationship between exposome and genome (70) and can also provide a biologically grounded framework to explain intergenerational transmission of stress (71,72).

### How Do We Get Closer to Causality?

A major motivation to study the exposome is to identify modifiable targets for intervention that are on the causal pathway to disease, with the notion that the exposome is in principle malleable. To that end, it is critical to address specificity, as highlighted above, both from the exposure and from the outcome perspective. However, acknowledging that environment is a dynamic complex of interactions that require an exposome approach and does not allow studying of individual exposures in isolation (e.g., trauma, prenatal cannabis exposure) may result in the unwanted situation whereby it becomes impossible to dissect specific targets in the exposome that may be on the causal pathway to a trajectory/outcome. A key priority is to apply causal inference frameworks that can inform mechanisms through which environment alters brain and behavior and allows for targeted interventions (73).

## Conclusions

To conclude, nongenetic factors play a key part in psychosis, and the developmental nature of PSD calls for deeper investigation of environmental origins. We propose that adoption of an exposome framework to capture the dynamic intertwined nature of environment should become an integral part of the study of psychosis etiopathology to complement psychiatric genomics research. To emphasize, despite the major advances in psychiatric genetic research and the increasing sample sizes of genetic studies, the effect sizes of individual genetic variation are much smaller than those of individual environmental stressors. When combined with the fact that environmental modification is much more readily achievable than genomic modification, the imperative for rigorous, comprehensive, well-powered, longitudinal exposome studies to guide new strategies for prevention and early intervention becomes even clearer.

The exposome framework requires us to investigate environmental exposures at multiple levels and consider both additive and interactive effects of exposures (rather than narrowing environment research to a single exposure), while accounting for the collinear nature of exposures. In addition, the developmental nature of psychosis and the fact that environmental exposures have more salient effects early in the lifespan during sensitive periods requires us to study the exposome in young developmental cohorts, sometimes years before the psychosis spectrum phenotype is crystalized. Relatedly, when studying developmental cohorts, it is critical to acknowledge key characteristics of youth psychopathology, such as nonspecificity and heterotypic continuity, and study trans-syndromal phenotypes as outcomes or trajectories. Guided by these key points for research design, we hope to shed some light on the exposomic, genomic, and epigenomic mechanisms underlying developmental trajectories of trans-syndromal phenotypes in the Youth-GEMs (Gene Environment Interactions in Mental Health Trajectories of Youth) study funded by the European Commission Horizon Program (Figure 2).

Finally, we reiterate our standpoint that while the diversity of study populations and differences in resources and cultures pose major challenges to harmonizing exposome research across cohorts, we suggest that adoption of the exposome research framework by researchers across the globe will help explain variability in developmental trajectories, enhance generalizability of findings, and advance our understanding of the variability observed on the path from developmental risk to resilience.
